# Patients’ Experiences of Treatment Burden After Bariatric Surgery—An Exploratory Study of Sex- and Gender Differences

**DOI:** 10.1007/s11695-025-08148-1

**Published:** 2025-08-07

**Authors:** Elin Bruto Winberg, Monique Heijmans

**Affiliations:** 1 University of Gothenburg, Gothenburg, Sweden; 2 Netherlands Institute for Health Services Research, Utrecht, Netherlands

**Keywords:** Obesity, Bariatric surgery, Treatment burden, Sex- and gender differences

## Abstract

**Background:**

As treatment for severe obesity, bariatric surgery results in permanent changes to the body and requires lifestyle changes that are often life-long. The problems and challenges that people experience after bariatric surgery have not been systematically studied in regard to sex- and gender differences, although literature suggests that sex differences do exist.

**Methods:**

To study the problems and challenges that people experience after bariatric surgery, the innovative concept of treatment burden (TB) was used. TB goes beyond the experience of symptoms and looks at the broader challenges that patients may experience such as physical, technical, logistical, and sense-making challenges. Using a literature review of qualitative studies as well as a short qualitative survey among Swedish patients that had undergone bariatric surgery, attributes of TB after bariatric surgery were explored as well as possible sex- and gender differences.

**Results:**

The results of the literature review showed that challenges related to making sense, mentally coping with the period after treatment, and technical aspects of coping with the aftermath of surgery were the most experienced among patients. Although quantitative studies showed clear sex- and gender differences in complaints and outcomes between men and women, these were not assessed in qualitative studies.

**Conclusions:**

TB as experienced by patients after bariatric surgery is a multidimensional concept that asks for a holistic approach of treatment after bariatric surgery. Although literature suggests sex- and gender differences in the personal experiences after surgery, more research is needed to be able to provide gender-sensitive care.

**Supplementary Information:**

The online version contains supplementary material available at 10.1007/s11695-025-08148-1.

## Introduction

Obesity is a chronic complex disease that can lead to an increased risk of type 2 diabetes, heart disease, affect bone health, and increase the risk of certain cancers [1, 2]. Aside from the medical impact, obesity has been shown to have a substantial financial and societal impact across countries despite differences in healthcare systems and income levels [3]. Prevention and treatment of obesity are therefore of high priority. The national and regional guidelines for obesity treatment in primary care in Sweden state that adults with a BMI ≥ 30 should receive care in the form of an individualized treatment plan, including diet, physical activity, and the support needed to start and maintain behavioral changes. For those with severe obesity, that is a BMI ≥ 40 or BMI ≥ 35 with comorbidities, pharmaceutical treatment or bariatric surgery is indicated [4, 5]. The two most common surgeries being performed worldwide are Sleeve Gastrectomy and Roux-en-Y Gastric Bypass (RYGB) [6]. Both types of surgery alter the gastrointestinal system in a way which results in a lower caloric intake and an alteration in hunger signals, among other changes [7].

The permanent physical changes, lifestyle changes, and adherence to dietary rules that come with bariatric surgery may lead to significant weight loss but can also cause mental stress and other struggles [8]. The effects of a changing body on mental health, possible malnutrition or vitamin deficiencies, susceptibility to addiction, and other adverse side effects following bariatric surgery have been highlighted in a number of studies [9, 10]. These effects are important to consider when providing care and support to this patient group, as these effects not only impact quality of life but may also hinder successful adherence to lifestyle recommendations. This is also acknowledged in the Swedish national guidelines [4]. They state that after bariatric surgery, a lack of a holistic view on health and treatment may not only lead to the absence of positive changes but possibly even result in negative outcomes. The national guidelines mention that long-term follow-up (either through primary- or specialized care) is needed after bariatric surgery and that multi-professional cooperation is necessary to ensure structured and regular follow-up care. The guidelines do not however go into details regarding recommended follow-up care after bariatric surgery [4], probably as there is a gap in knowledge about which aspects of coping with the aftermath of bariatric surgery are especially important to patients and need further attention.

### Treatment Burden and Bariatric Surgery

**Fig. 1:**
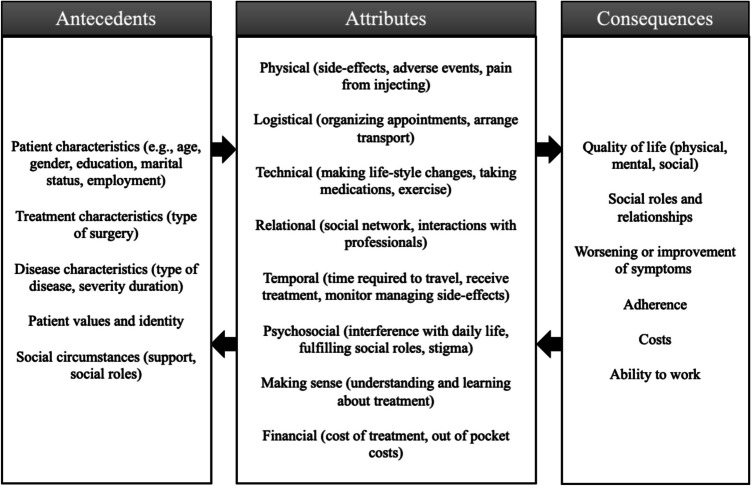
Theoretical model of TB used to describe the results. Source: Gallacher et al. [11]; Sav et al. [13]

This study aims to better understand the experiences of people that have undergone bariatric surgery, and for this purpose we focus on a relatively new and innovative concept in care and research: treatment burden (TB). TB refers to the problems and challenges that people with chronic conditions encounter and the self-care practices they must perform to follow the complicated management strategies that have been developed for these conditions [11]. It is a multidimensional concept consisting of different aspects of treatment by which patients feel challenged in their daily lives and is based on the experiences of the patients themselves [12]. Gallacher et al. [11] and Sav et al. [13] developed a generic conceptual model, defining the aspects or attributes of TB, based on a number of studies in different chronic conditions (see Fig. 1). This model makes a distinction between logistical burdens (e.g., organizing appointments or visits from health professionals, organizing rehabilitation, arranging transport), technical burdens (e.g., making lifestyle changes, taking medication, doing exercises), relational burdens (e.g., enrolling family and friends and health professionals for support, initiating interactions with people who had the same experience), and making sense burdens (e.g., conceptualizing problems, understanding and learning about management, knowing when to seek help, and differentiating between treatments or lifestyles) for example [11]. TB is not a static concept and will differ among individuals and stages of treatment. Therefore, the TB model also includes antecedents (e.g., patient characteristics such as sex and gender, treatment and disease characteristics, and social circumstances) that may influence the treatment burden (attributes) experienced as well as possible consequences (e.g., quality of life, social roles and relationships, costs, adherence, ability to work, and improvement or worsening of symptoms) [13]. The concept of treatment burden adds a distinct and valuable layer to existing studies on personal experiences after surgery, including bariatric surgery. While many qualitative studies explore emotions, satisfaction, or identity shifts post-surgery, treatment burden brings in a more systematic, health-services-focused lens. It provides actionable insights about the work of being a patient and as such makes it a powerful tool for patient-centered care [14]. It is unclear if the TB model as described in Fig. 1 also fits the experiences of patients that have undergone bariatric surgery. Burden may relate to the surgery itself as well as the treatment and lifestyle changes after surgery. An in-depth study of TB in this patient group can help inform caregivers and physicians on how to optimize treatment with minimal burden to patients. So far, there is no synthesis of knowledge on attributes of TB in patients with bariatric surgery and its antecedents or consequences.

### Sex- and Gender Differences in Bariatric Surgery

Obesity and severe obesity are globally more common in women than in men, although in Sweden, it is the other way around where 18.1% of men and 14.4% of women are obese [15]. Also, the utilization of bariatric surgery differs between sexes as 80% of those undergoing bariatric surgery globally are female [16]. Besides differences in prevalence for obesity and bariatric surgery, there are also sex differences both preoperatively and postoperatively. Men tend to have higher preoperative BMI, more comorbidities, and experience postoperative complications to a greater extent than women. Despite women losing more excess weight and having less comorbidities and complications postoperatively compared to men, some studies suggest that they have lower rates of satisfaction after surgery [17, 18]. A study by Kochkodan et al. [18] found that women have lower body image than men, even when having objectively greater results after bariatric surgery.

### Treatment Burden and Sex and Gender

There are several quantitative studies that have examined sex differences after bariatric surgery in complaints and outcomes [16] [19], but there is a lack of literature exploring personal experiences after bariatric surgery as it relates to sex and gender. This knowledge gap calls for further research within this area. When studying sex- and gender differences in TB, a distinction should be made between the terms sex and gender. Sex can be described as biological attributes such as chromosomes or hormone levels, whereas gender may refer to socially constructed roles and behaviors of men and women for example [20]. Sex and gender interact with biological, social, and economic determinants of health and as such may contribute to the different outcomes found for men and women after bariatric surgery. Differences between the sexes may be related to the differences found in physiological outcomes of bariatric surgery (such as complications, weight loss or resolution of comorbidities) [16], whereas the psychosocial differences such as body image and satisfaction rates could be explained by gender roles [21]. Insight into sex- and gender differences in treatment burden may contribute to more gender-sensitive care and as such improve care regardless of gender.

The lack of literature exploring the personal experiences of patients after bariatric surgery (TB), as well as the limited understanding of sex- and gender differences in this context, elucidates the need for further research within this area. The primary goals of this study are therefore (1) to explore patients’ experiences of TB after bariatric surgery and (2) to see whether they differ between men and women, and according to gender roles. In this way, we hope to better understand TB after bariatric surgery and the reasons behind differences in outcomes between men and women with the hope of informing future care practices.

## Methods

### Study Design and Concepts

To explore the concept of TB in patients after bariatric surgery and search for possible sex- and/or gender differences within this group, a mixed-methods approach was used. This includes a literature review of available qualitative literature in the past 4 years as well as a short survey to find aspects of TB after bariatric surgery as perceived by men and women in Sweden. The choice of the 4-year period and of conducting the survey only within a Swedish context was based on the time limitations for the study. Within this study, the theoretical model as described by Gallacher et al. [11] and Sav et al. [13] was used as a framework (see Fig. 1) to operationalize TB. Our aim was to describe the different attributes of TB as experienced by patients after bariatric surgery. As the focus was on qualitative studies and the personal experiences of patients, we did not expect to find much information on antecedents and consequences, also defined in the framework. However, information accidently encountered was also reported. All findings from the literature and the survey were systematically organized in extraction tables (see Appendix 1 and 2). The extraction tables include information on the country in which the study was conducted, possible antecedents (which may for example include sex, gender, age, type of surgery, time since surgery, marital status, and income), type and aspects of TB attributes, reported consequences if available, a summary of main findings and sex- and gender differences.

### Literature Review

#### Search Strategy and Study Selection

Because of the limited timeframe of this study, the literature review was built on a recent systematic review of qualitative studies by Li et al. [22] covering the period January 2011 to April 2021, where the personal experiences of those that have undergone bariatric surgery were explored. An update of this original search, using the same methods and search strategies, was performed for the period of April 2021 to October 2024, with the aim of adding the most recent papers on patient’s experiences after bariatric surgery to those identified by Li et al. [22]. The review by Li et al. [22] was chosen as a foundation for this study as it includes articles with a wide range of experiences after bariatric surgery. For the additional search, the same search string and MeSH keywords were used as in the review by Li et al. [22]: (“Bariatric Surgery” OR “Gastric Bypass” OR “Weight Loss Surgery” OR “Gastric Sleeve” OR “Roux-en-Y Gastric Bypass” OR “Metabolic Surgery”) AND (Diet* OR Eat OR Food) AND (Experience* OR Perception* OR Attitude* OR “Support Need*” OR Support) AND (“Qualitative Research” OR “Qualitative Studies” OR Interviews OR NVivo). TB was not included in the search string as an initial quick search revealed no qualitative studies that utilized the concept of TB in relation to bariatric surgery. By not including the rather specific term of TB in the search string but using broader terms such as “Experience”, “Perception” or “Attitude” instead, we could ensure that a broad range of qualitative studies that explore experiences after bariatric surgery were included. These experiences could then be ordered according to the different attributes of TB if applicable. The additional search was conducted through PubMed. Inclusion criteria were papers of qualitative studies describing patient experiences after bariatric surgery, adults (≥ 18 years), published between April 2021 and October 2024 in English or Swedish. Although Li et al. [22] mainly focused on dietary experiences after bariatric surgery in the description of their results, our focus was broader and included any experience after bariatric surgery that fits one or more of the attributes of TB. When re-reading the papers selected by Li et al. [22], we therefore aimed to capture all experiences mentioned and not only those related to diet.

### Survey

A short survey regarding patient experiences after bariatric surgery was also distributed in two Swedish Facebook support groups for bariatric surgery patients. The survey was meant as an extra validation of the TB aspects that would be found in literature. The survey contained one main question of what the participants experience as the most important challenges after bariatric surgery. Participants could mention up to 10 aspects of TB and were asked to rank them according to the extent to which they impact their daily lives, starting with the aspect with the greatest impact. To help them frame their thoughts, some examples of challenges were given such as diet, interactions with caregivers, social interactions and stigma, adapting to treatment in everyday life or at work, information needs, taking medications and supplements, and financial consequences (representing different attributes of TB). Also, some background information was inquired, namely, sex, age, type of surgery, and time since surgery. An added textbox to the question of sex allowed respondents the possibility to clarify their sex and gender.

### Analysis of Results

Findings from the literature review and survey were extracted by the first author, and 20% of the studies and all answers from the survey were checked by the second author. Subjective experiences from the included articles and survey answers were categorized according to the attributes of the conceptual framework [11, 13]. Disagreements were discussed. When a result or subjective experience agreed with more than one attribute of TB, the most obvious aspect was chosen for categorization. As this is a subjective judgement and attributes are interrelated, doubts were discussed until agreement was reached. Results from the extraction table were then quantified to determine which attributes of TB were most and least common as well as what these attributes consisted of. Quotes from the survey were used to illustrate and confirm the findings from the literature.

## Results

**Fig. 2:**
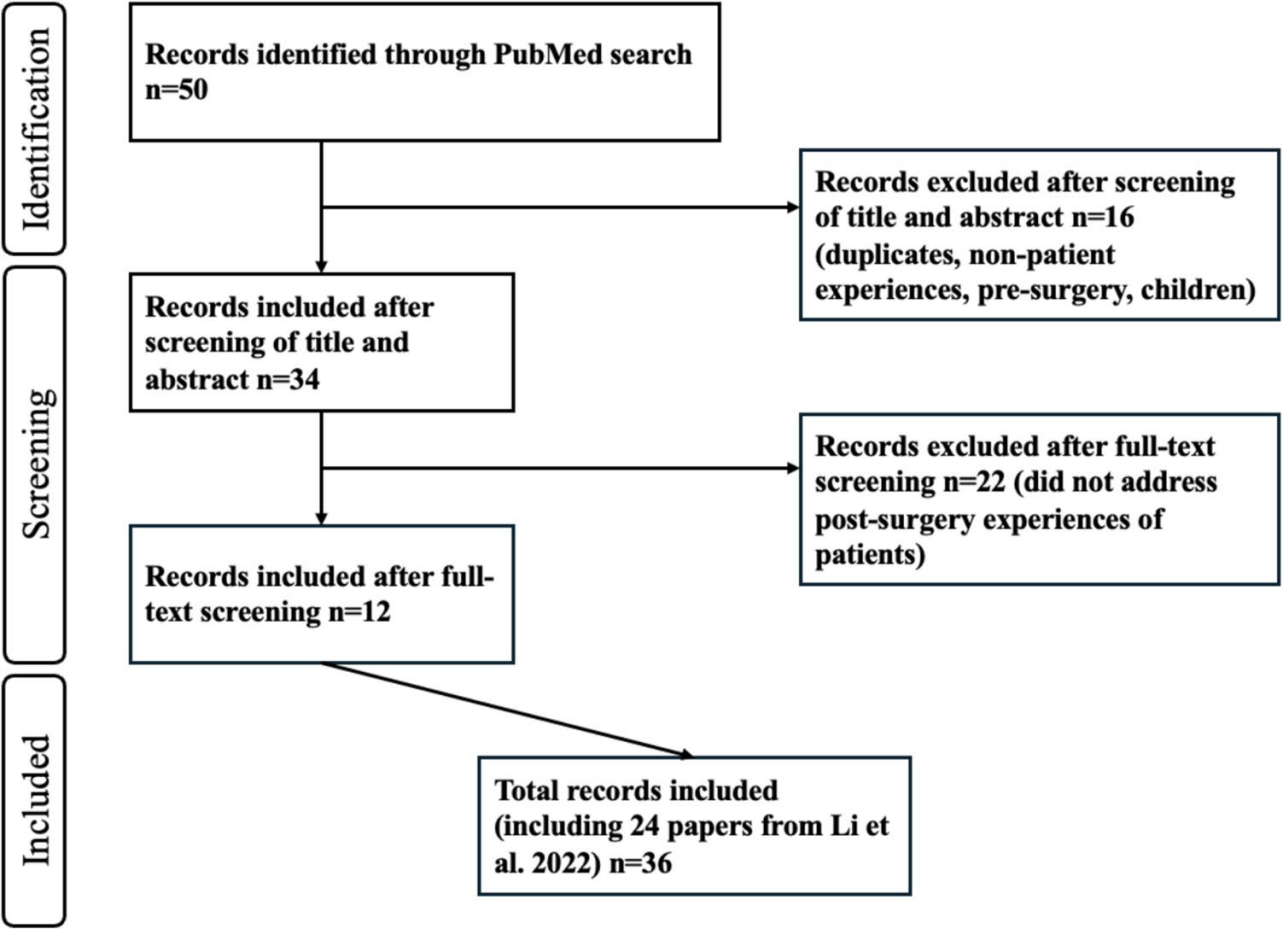
Flowchart for selection of studies of updated search

By the updated search, 50 studies were found that were of possible interest for this study. Based on title and abstract, 16 studies were excluded because they did not meet the inclusion criteria of this study. Of the remaining 34, full texts were read; 22 studies were further excluded mostly because they did not capture the personal experiences of patients after bariatric surgery, resulting in 12 included studies from the additional search (see Fig. 2 for the flow chart of the additional extraction process, based on Page et al. [23]). In total, 36 studies were included in this literature review; 24 from the review written by Li et al. [22] and 12 from the additional updated search we conducted. Of the 36 studies, most studies were conducted in the USA (*n* = 8) followed by the UK (*n* = 6), Sweden (*n* = 5), Norway (*n* = 4), China (*n* = 3), and Australia (*n* = 3). From Portugal, Denmark, Canada, Turkey, New Zealand, the Netherlands, and Belgium, one study each was included. Sample sizes within the qualitative studies varied between 3 and 32 and the majority of the participants were women, with 77% of the total number of participants being women. Most participants underwent RYGB (Roux-en-Y gastric bypass), but sleeve gastrectomy and gastric banding were also common. Time since operation varied between 5 months and > 15 years.

### Aspects of Treatment Burden

**Fig. 3:**
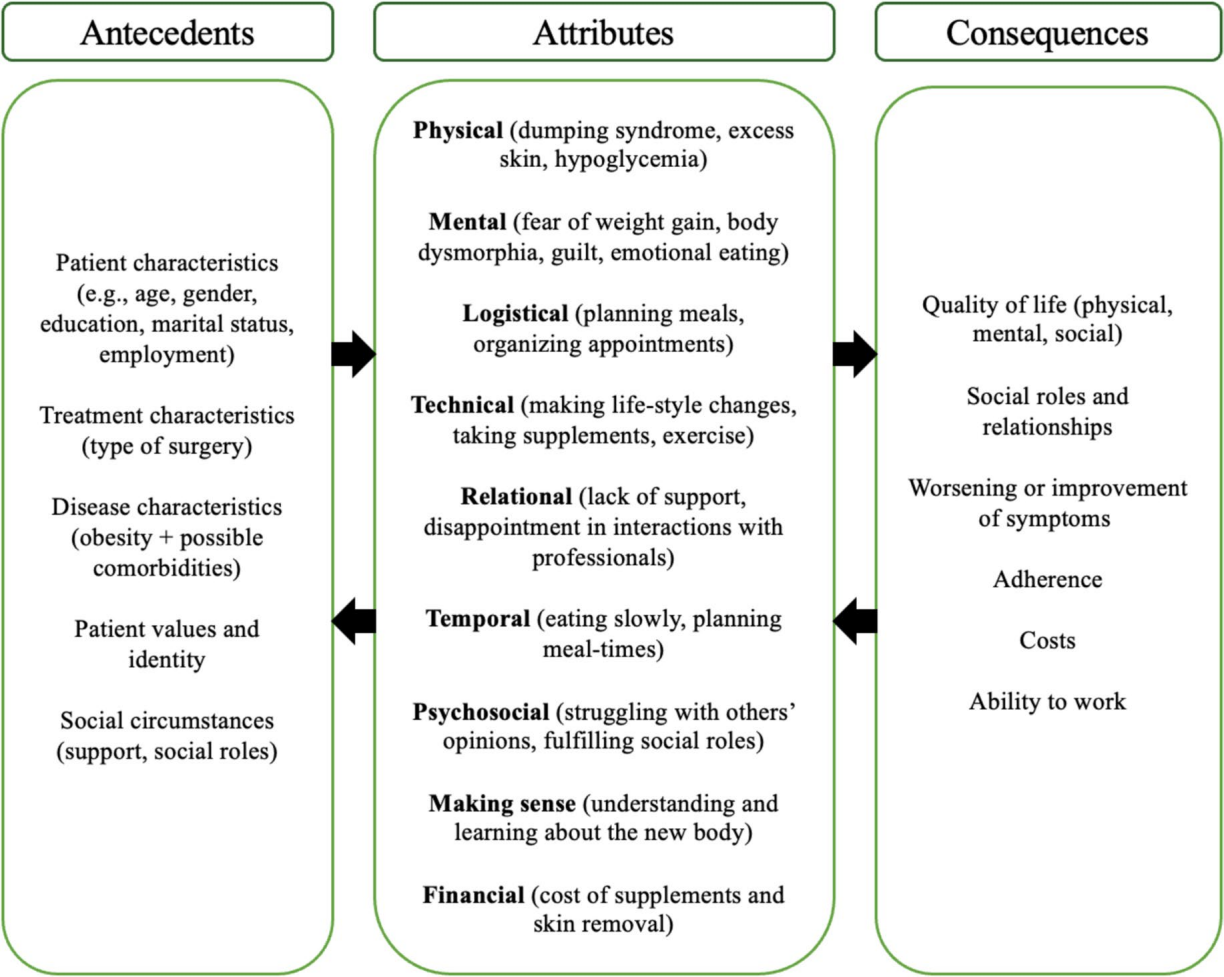
Conceptual framework of TB adapted for bariatric surgery

Table 1 gives a summary of the aspects of TB encountered in the 36 studies included in this literature review. Full extraction tables can be found in Appendix 1 and 2. Regarding the attributes of treatment burden, aspects belonging to the attribute of *making sense* were found in 29 of the 36 studies, making it the most recurring TB attribute. This was followed by aspects belonging to the *mental* and *technical* attributes which appeared in 28 articles each. The least common attribute was *financial*; only 5 of the 36 studies mentioned aspects of TB belonging to this attribute. There were a number of aspects of TB found related to the psychological consequences of coping with the period after bariatric surgery that did not fit the attribute categories, as described in Fig. 1. Therefore, a new *mental* attribute was added, resulting in a slightly adapted model compared to the model in Fig. 1 for bariatric surgery. This adapted model is shown in Fig. 3. During extraction, it appeared that the results of the included studies were quite similar and confirmed each other, providing evidence for saturation of data.

**Table 1:** Aspects of TB attributes found in literature review (*n* = 36)

TB attribute	*n* (%)	Main aspects addressed in literature review
Physical	24 (67%)	• Dumping syndrome and food intolerances • Excess skin after weight loss leading to infections • Hypoglycemia • Constipation • Malnutrition • Pain • Hair loss • Reflux • Increased alcohol sensitivity
Mental	28 (78%)	• Fear of weight gain and the stomach pouch stretching • Body dysmorphia and struggling with body image due to the changing body and excess skin • Feelings of guilt, anger and blaming oneself after a dumping episode • Food addiction and emotional eating being replaced with alcohol • The mental effects of not losing the desired amount of weight, feelings of disappointment and regret after surgery • Psychological stress due to food choices being severely restricted • Still feeling obese • Experiencing mental hunger despite being physically full
Logistical	13 (36%)	• Planning meals and monitoring food consumption • Having regular planned meals to avoid dumping syndrome • Planning for trips and events involving food • Eating enough protein • Adhering to follow-up visits to prevent malnutrition • Support group meetings being too far away or not having access to childcare • Lack of public transportation in rural areas leading to missed follow-up appointments • Patient occupation interfering with adhering to post-surgery guidelines
Technical	28 (78%)	• Making permanent lifestyle changes to eating habits and physical activity and the struggles of adhering to these changes • Taking daily supplements to prevent malnutrition • Adhering to dietary restrictions to minimize risk of dumping syndrome, hypoglycemia and maintain weight loss • Trying to create good habits in the ‘honeymoon phase’ to adhere to in the ‘work’ phase of maintaining weight • Lifestyle changes to minimize physical discomfort, such as smaller portions, eating slowly and chewing more
Relational	19 (53%)	• Disappointment in follow-up care by care providers. It mainly consists of monitoring and weighing, whereas emotional health is not being paid attention to. Wishing for longer term support and psychological follow-up care • Insufficient information from care providers, lacking information about adherence to diet and portion sizes long term • Lack of support from family, friends and partners • Avoiding social eating due to food restrictions, leading to isolation • Ending relationships due to the negative impact they had post-surgery (e.g. negative comments or negative impact on eating behavior)
Temporal	14 (39%)	• Planning meals, eating slowly and often • Managing side-effects such as hypoglycemia by planning food intake • Taking time for physical activity • Inconvenient meeting times for support groups • Avoiding follow-up appointments as they take up too much time • Dosing schedules for supplements
Psychosocial	24 (67%)	• Struggling with other people’s opinions about the changing body, scars and excess skin. Feeling self-conscious when undressing in front of other people or worrying about sexual attractiveness • Fear about others’ opinions about weight gain • Grief regarding not being able to enjoy the same meals as others socially • Fear of dumping syndrome when at work or out in public. Learning to be okay with leaving food on the plate despite what others might think, to avoid dumping syndrome • Struggling with other’s opinions about having bariatric surgery • Struggling with the role of being a mom but also taking care of oneself • Ignoring eating restrictions to appear like a ‘normal man’ • Support from friends and family sometimes being stigmatizing and discouraging. Finding additional attention and comments about weight to be uncomfortable
Making sense	29 (81%)	• Getting used to the new body, both how it looks and how it feels • Being surprised and not having knowledge about certain aspects and outcomes after surgery (e.g. experiencing a change in physical restrictions or increased alcohol sensitivity) • Learning to listen to the body’s fullness cues and tolerances, relearning how to eat • Believing that surgery would be a quick fix and having to come to terms with the effort having to be put in after surgery • Lack of health literacy making it difficult to adhere to post-surgery guidelines • Learning that the surgery is not a cure for emotional or disordered eating • Dealing with unrealistic expectations not being met • Viewing dumping syndrome as a positive consequence that prevents weight gain
Financial	5 (14%)	• The cost of excess skin removal • The cost of nutritious foods and supplements

The survey was filled in by 44 respondents of which 43 were women (98%). Five respondents were excluded as they did not give information related to the question asked. The most common aspect mentioned by respondents in the survey had to do with *technical* challenges*,* which was found in the experiences of 29 respondents. The least common attribute was *financial* challenges*,* which was not found in any of the respondent’s experiences. Results from the survey confirmed the experiences found in the literature review and provided no new information. Quotes from respondents to the survey were therefore used to illustrate the main findings from the literature.

Within the *physical* attribute of TB (side-effects and adverse events etc.) dumping syndrome, food intolerances, excess skin after weight loss, hypoglycemia, and constipation were mentioned most often [24–28]. Respondents of the survey also mentioned excess skin and the weight of carrying it around. They additionally mentioned the physical restrictions and the difficulty of drinking water in bigger amounts as it may result in abdominal pain.

Aspects of the *mental* attribute (psychological impact of treatment), an attribute that was added to the framework as it was frequently mentioned, included a range of fears and feelings of guilt. Common aspects found in the literature review were fear of weight gain, body dysmorphia and body image issues in relation to the changing body and excess skin [29–33]. Also, feelings of guilt when not eating according to recommended guidelines and having a dumping episode, replacing emotional eating with alcohol abuse, and psychological stress due to food choices being restricted or limited were often mentioned [25, 26, 34–36]. Respondents of the survey confirmed these findings by mentioning the “fear of weight gain” (Resp. #34) and the struggle to “…not get too unhealthy thoughts e.g. getting stuck [in] that I have to lose weight” (Resp. #39).

Challenges related to the *logistical* attribute (e.g., planning and organizing related to treatment) mentioned in literature and survey answers included planning meals and remembering to “bring snacks and food when I am not at home” (Resp. #30), regular mealtimes, planning for trips or social situations involving food, eating enough protein, and adhering to follow-up visits with care providers [37–41].

Some of the most recurring aspects related to *technical* challenges (making lifestyle changes, taking medications and supplements etc.)*.* This included challenges such as adhering to permanent lifestyle changes to diet and exercise, taking supplements daily, and adhering to dietary restrictions to avoid side effects such as dumping syndrome or hypoglycemia [42–47]. Remembering to “…eat slowly and often” (Resp. #31) and to “take my medications and vitamins every day” (Resp. #30) were the most common aspects of the technical attribute (an attribute which was present in 66% of the respondent’s answers and therefore the most common) within the survey.

Experiences within the *relational* attribute (e.g., social network and interactions with professionals) included disappointment in follow-up care from care providers and wishing for more psychological follow-up care, lack of support from family and friends, avoiding eating in social situations which leads to isolation and changes to relationships post-surgery [28, 35, 48–52]. Responses to the survey regarding aspects of the relational attribute highlighted lack of support, and specifically psychological support, from care providers after surgery as one respondent shared that “I had to cope with my food addiction on my own without anyone to talk to who helped me with mental tools” (Resp. #16).

The time it takes to plan meals, to eat slowly and regularly, perform physical activity and attend follow-up visits are all challenges related to the *temporal* attribute (time allocation for treatment and managing side-effects etc.) that were found among participant’s experiences in the literature review [24, 31, 43, 53]. Difficulties with time management regarding regular, and an increased number of meals during the day post-surgery, were the most common aspect of the temporal attribute within the survey answers.

Experiences related to the *psychosocial* attribute of TB (e.g., interference with daily life, fulfilling social roles and stigma) that were found in the review included struggling with other’s opinions regarding bariatric surgery or the changing body, grief related to not being able to enjoy the same food socially and struggling with the role of being a mom but also taking care of oneself [36, 45, 48, 50, 54]. Results from the survey validated the findings regarding aspects of the psychosocial attribute of TB, as struggling with other people’s opinions was the most common experience among respondents: “Explaining to all people how I was able to lose weight so quickly. I know that many people don't like this kind of surgery because they think it's an easy way to go, which is completely wrong” (Resp. #11).

Aspects of the most common attribute found, *making sense* (understanding and learning about treatment, conceptualizing problems etc.), included getting used to how the new body looks and feels, learning to listen to the body’s new fullness cues and food intolerances, relearning how to eat, being surprised and learning about certain aspects and outcomes after surgery (e.g., experiencing a change in physical restrictions or increased alcohol sensitivity) [30, 35, 44, 55–57]. Within the survey, the attribute of making sense was also found in aspects of relearning and understanding the new body, as well as adapting the mind, after surgery: “The hardest part has been rewiring the brain. The tool in the body is there but not in the head.” (Resp. #16).

Finally, *financial* (cost of treatment, out of pocket payments) was the least common attribute and challenges found included the cost of excess skin removal as well as the costs of supplements [32, 53]. There were no aspects of the financial attribute found within the survey answers. Although the financial attribute was the least common, it was present in patient experiences and therefore all attributes of the TB model were found within the literature review.

### Comparison Survey and Literature Review

Results from the survey confirmed the findings in the literature review as aspects of the different TB attributes, including the added *mental* attribute, were similar in terms of common experiences. Data from the review and survey were collected based on different methods and questions, but both elucidated the range of aspects of TB attributes that are present after bariatric surgery.

### Sex- and Gender Differences

Findings in relation to sex- and gender differences in treatment burden after bariatric surgery were very limited as few of the included papers utilize the concepts or explicitly explore the differences between men and women. In a paper by Groven et al. [24], men’s experiences in regard to making sense of their new body after bariatric surgery were explored and related to the concept of hegemonic masculinity. This is illustrated by one participant mentioning that he will ignore food restrictions to appear like a “normal man.” In another paper, Groven [36] inquired into the experiences of women after bariatric surgery and how these relate to the sociocultural world and notions of womanhood. These papers do not compare the experiences of men and women but contribute to experiences related to the concepts of gender rather than sex, which is currently the most common in literature examining experiences after bariatric surgery. In additional articles, the concept of gender is not explicitly mentioned but it is possible to detect experiences that are related to gender roles. For example, feelings of guilt in relation to the roles of being a wife and mother after suffering complications post-surgery [54]. As 98% of the survey respondents were women, it is not possible to deduce any sex- and/or gender differences from these responses.

### Antecedents and Consequences

As the focus of this study was on the personal experiences of those that have undergone bariatric surgery, the attributes of TB were the focal point of analysis. However, when applicable, antecedents and possible consequences were also extracted from the data to create a fuller picture of TB as it relates to bariatric surgery. All papers in the literature review included information on participants’ sex or gender, a patient characteristic that as an antecedent may impact TB, but this characteristic was not used in the analysis as it was not part of the results. Another antecedent that may impact TB is the type of bariatric surgery, which was disclosed in most included papers, but based on the available data it is not possible to conclude how this impacts TB. Based on the aspects of TB that were found among participant’s experiences; some possible consequences were also identified. Most commonly, quality of life, adherence, and social roles were impacted by the challenges experienced by patients.

## Discussion

The aim of this study was to explore which attributes of treatment burden are experienced by those that have undergone bariatric surgery, and to see if these attributes differ in regard to sex and/or gender. A mixed-method study was conducted consisting of a literature review and a short qualitative survey to provide insight into the aspects of TB as experienced by patients after bariatric surgery. Although this was a first explorative study on the TB concept in a short timeframe, results from the literature review clearly highlighted a broad and diverse range of challenges that patients experienced and had to deal with after bariatric surgery with challenges in relation to making sense of their experiences, coping mentally with the aftermath of surgery, and technical challenges related to diet and lifestyle as the ones most experienced by patients. The results were confirmed by the survey among Swedish patients that underwent bariatric surgery. In this survey, logistical and physical burdens were also mentioned often.

The term treatment burden was not explicitly mentioned in any of the included studies in the literature review, and the concept of TB was instead applied to the results of patient’s subjective experiences after bariatric surgery. This study is therefore only an initial exploration of TB and how it relates to bariatric surgery, and more research should be conducted to accurately and systematically provide evidence for the relevance of TB in relation to bariatric surgery. Treatment burden is a relatively new concept and underexplored in regard to bariatric surgery, but we believe that the concept can add a distinct and valuable layer to existing studies on personal experiences after bariatric surgery. While many qualitative studies explore emotions, satisfaction, or identity shifts post-surgery, TB brings in a more systematic, health-services-focused lens providing better cues for holistic person-centered care. In comparison with research on TB in other diseases [11, 13, 58], we found that aspects of mental struggles related to surgery were relatively present among people that underwent bariatric surgery. Mental as an attribute was not included in the initial framework developed by Gallacher et al. [11] and Sav et al. [13] but should be added as an aspect of TB for bariatric surgery as the treatment’s impact on the mental health of participants became apparent from both the literature review and the survey. Therefore, this study clearly shows that treatment burden after bariatric surgery is a multidimensional concept and encompasses more than physical complaints and lifestyle changes. It asks for a holistic approach and a multidimensional assessment by healthcare professionals when supporting or treating patients after surgery in which mental, sense-making aspects and social aspects are equally important. Not only in the period directly after surgery but also long-term, as bariatric surgery is a lifelong treatment for obesity [4].

Despite there being very limited findings of sex- and gender-related differences in this qualitative study, we still consider conducting an exploration of this important as quantitative research has shown that differences do exist. Sex differences are often mentioned in quantitative literature on bariatric surgery, describing for example differences between males and females in regard to risks of surgery complications and outcomes [19, 59]. Also, differences pertaining to gender are in some studies implicitly mentioned in relation to bariatric surgery [17, 18], although these studies do not delve deeper into the differences of subjective experiences. Findings from these quantitative studies made us curious about sex- and gender differences in the personal experiences of men and women after bariatric surgery as a possible explanation for these quantitative differences, but very few studies made a distinction between sex and gender. However, the few implicit gender differences, or experiences related to different gender roles, that were found are worth exploring further. For example, participants explained that the role of being a mother is important to them and that it is difficult to find time for oneself after surgery [35]. This can be related to the antecedent of being a woman (gender) as well as the psychosocial attribute of TB; in how fulfilling the social role of being a mother is impacting the ability to take care of oneself post-surgery and therefore increasing TB [13]. Previous quantitative research has indicated that women have lower body image compared to men after bariatric surgery [18], but research explicitly exploring gender differences based on patient experiences is needed to further understand these differences. Men are underrepresented in qualitative health research [60], not only in relation to bariatric surgery, which further demonstrates the need for this research. A paper by Groven et al. [24] explores men's experiences of making sense of the changes to their body after bariatric surgery and relates this to the concept of hegemonic masculinity. Although the paper does not examine the differences between men and women, it elucidates the fact that men’s experiences after bariatric surgery may be different and relate to the concept of masculinity and gender, which needs further exploration. The lack of explicit mentioning of sex and gender differences in the data included in this study limits the possibility to draw further conclusions considerably. However, it does highlight the importance of future research (specifically qualitative) exploring these differences to aid in the development of more gender-sensitive care for those that have undergone bariatric surgery.

The difficulties of inferring sex- and/or gender differences in TB after bariatric surgery could be due to most papers in this literature review not utilizing the concepts or comparing experiences after bariatric surgery between men and women. It is also important to note how sex and gender is operationalized when used within research. When discussing physical attributes of TB, such as hormonal changes and their impact on the body after bariatric surgery, sex may be the preferred term to use in regard to biological differences. When discussing psychosocial aspects, such as the difference in burden due to gendered parental roles, gender may better communicate the implications. If these terms are used interchangeably or used without discussing the implication of sex and associated gender, nuance can be lost in the analysis [20]. The differentiation of these terms could therefore have a great impact on recommendations and consequences of research. For example, physiological differences between sexes due to anatomical differences may lead to one outcome in recommendations after bariatric surgery based on biological differences or lack thereof [59]. Regarding differences in psychological wellbeing post bariatric surgery, outcomes may depend on if the differences are attributed to sex or gender. For example, the difference in aspects of the psychosocial attribute of TB, such as the added burden of the social role of being a mother, which is connected to gender roles. By reducing the results to sex differences, which indicates an inherent difference between males and females, the structural issues of gender differences after bariatric surgery may not be tended to (e.g., increased childcare support and gender equity).

Due to the focus of this study being the attributes of TB, we cannot say much about antecedents and consequences. However, seeing that sex- and/or gender as an antecedent may impact the attributes of TB being experienced (and thereby the following consequences), this is an area that needs to be explored further. This also includes other antecedents such as type of surgery as well as social circumstances and how they relate to social roles based on gender. Due to the qualitative nature of this study and small sample sizes this was not possible.

Aside from sex- and gender differences, the findings regarding patient’s experiences of TB after bariatric surgery show that much can be done in general to minimize the burden for both men and women. Two of the most common aspects of TB being the attributes of making sense and mental show that psychological aspects are important to consider even further both before and after surgery. Participants mention being surprised by certain aspects of their new body after surgery, having a lack of information and needing further psychological support as bariatric surgery only alters the gastrointestinal system and not the individual’s relationship with food. This is also illustrated in relation to gender by the fact that some men hesitate regarding asking for help when experiencing severe side effects [24] and women struggling more psychologically after bariatric surgery compared to men [18]. Previous reviews have already shown that further monitoring of psychological effects after bariatric surgery is needed to understand the mental effects on patients [61]. Increased attention to these psychological effects as well as giving patients more information regarding long-term effects could therefore contribute to minimizing TB, irrespective of sex or gender.

### Limitations and Reflexivity

The concept of treatment burden has not yet been utilized extensively in relation to bariatric surgery and this study therefore only contributes with an initial attempt to connect the areas of research, with the hope of continuing to provide further evidence of relevance. Also, as an exploratory study, this study mainly explored the attributes of treatment burden, whereas future research should aim to utilize the whole conceptual model (including antecedents and consequences) to attain an understanding of the different part’s impact on experiences of TB after bariatric surgery.

Due to time limitations, the survey only included respondents from Sweden. To get a representative image of TB as experienced by patients globally, the scope of respondents should be broadened in future research. This is specifically relevant as countries and cultures differ in dietary customs and social roles for example and this may impact experiences of TB. The lack of men participating in qualitative health research also contributes to the difficulties of reaching a full understanding of sex- and/or gender differences. It should be noted that even though the survey did confirm the findings of the literature, only one respondent to the survey was male (2% of the total sample). This could be explained by the fact that men are less likely to be in a support group [30]. Future research should strive to include a sample that is representative of the population to ensure that the experience of men who have undergone bariatric surgery is represented. It is therefore important to take this into account for future participant recruitment and data collection as the lack of a representative sample can limit the analysis. Additionally, the lack of explicit mentioning of sex- and gender differences in the papers included in the literature review significantly limits any conclusions that can be drawn from this. This, together with differences found in quantitative studies, does however elucidate the importance of future research within this area.

Out of the 36 articles included in the literature review, 24 were included based on a previous search performed by Li et al. [22] As the review by Li et al. [22] mainly focused on dietary experiences after bariatric surgery, our literature review may therefore be missing articles including a broad spectrum of experiences from articles published between 2011 and 2021 (the dates of articles included in the previous search). Also, the additional search for the literature review was conducted only in PubMed which could result in relevant articles missing from the included data. Future research may therefore benefit by conducting a larger search within the data available to ensure that all relevant papers are included. Performing a scoping review for example makes it possible to more systematically identify gaps in content and methods of studies aimed at gaining insight into the experiences of men and women after bariatric surgery. Within the timeframe of this study, this was not feasible. The data obtained from the articles included as well as the data from the survey did not contain information about the experiences of transgender individuals or those with a non-binary gender identity. Therefore, we can only comment on the possible differences between cisgender men and women and recommend that future research includes a more diverse population.

### Recommendations

Based on the results of this study, we offer recommendations both for future research but also for practices within bariatric care. Regarding research, even though sex differences in bariatric surgery prevalence and outcomes have been found in quantitative research, there is a lack of qualitative studies exploring patient experiences after bariatric surgery in relation to sex and gender. We therefore recommend that future research utilizes both quantitative and qualitative methods to further explore how sex and gender may impact aspects of TB after bariatric surgery. We also recommend that the concepts of sex- and gender are clearly defined within the scope of the research and to explain how the concepts are used in relation to TB, for example when referring to anatomical differences or social roles. It is also important to ensure that men are represented in health research and to broaden the scope for recruitment (beyond support groups for example). The qualitative studies included in this literature review did not explore experiences regarding different bariatric surgery methods, but this should also be explored further in relation to TB to understand how surgical method impacts treatment burden experiences. Also, even though financial aspects were mentioned least among participants, in comparison to other TB attributes, it is still an area that should be examined further through research as not only bariatric surgery itself may be financed out of pocket but also considering supplementation costs for example. The literature included in the review also included participants with varying time since surgery, and future research should explore how TB varies through the post-operative period and throughout the rest of the life course. Regarding care practices, this study elucidated the need for more focus on the psychological effects of bariatric surgery. The TB model is broad and was useful in categorizing and understanding experiences after bariatric surgery, specifically shedding light on the mental aspects and the aspects of making sense of the changed body. It is therefore important that the future development of bariatric care employs a holistic view that not only focuses on changes to the physical body and lifestyle, but also to the long-term mental health of patients.

## Supplementary Information

Below is the link to the electronic supplementary material.

Supplementary file1 (DOCX 68 KB)

## Data Availability

All data from the literature review supporting the findings of this study are available within the paper and its Supplementary Information (see all papers in Appendix 1 and 2). The survey data that support the findings of this study are not openly available due to reasons of sensitivity and are available from the corresponding author upon reasonable request. Data are located in controlled access data storage at the University of Gothenburg.
