# Supplementary material for: Patients’ Experiences of Treatment Burden After Bariatric Surgery—An Exploratory Study of Sex- and Gender Differences
Source: Obes Surg. 2025 Aug 7;35(9):3430–42. doi: 10.1007/s11695-025-08148-1 (PMC12457569; doi:10.1007/s11695-025-08148-1)
Supplement: Supplementary file 1 — Supplementary file1 (DOCX 68 KB) [file 11695_2025_8148_MOESM1_ESM.docx]

**Appendix 1. Extraction table for papers from the review of Li et al. 2022**

| **Paper** | **Country** | **Ant: Sex, x̅ age (range), surgery, time since** | **Type of TB attributes** | **Aspects of TB attributes** | **Cons.** | **Main findings** | **Sex and/or gender diff** |
| --- | --- | --- | --- | --- | --- | --- | --- |
| Engström & Forsberg, 2011 | Sweden | 16(12 women)  36.8 (24-44)  Not reported  1-2 years  Marital status, employment, student and sick leave | Physical  Technical  Making sense  Psychosocial and mental | Dumping syndrome (however reported by participants as a positive side-effect) and physical eating restrictions – Also excess skin  Making permanent lifestyle changes  Experiencing a change in physical restrictions and not having information about long-term effects and changes after surgery  The mental effects of the changing body. Still feeling obese and struggling with external opinions about surplus skin | Adherence  Quality of life | Patients wished for deburdening through control over eating before surgery and experienced this control 1 year after surgery (for example through physical restrictions). 2 years post surgery these restrictions started to fade and participants feared weight gain. | N/A |
| Benson-Davies et al., 2013 | USA | 24(24 women)  51.8  (±10.5)  RYGB  At least 2 years  Other characteristics in a previous paper?? | Technical  Relational  Temporal  Logistical | Making lifestyle changes to eating habits and physical activity  Lack of support from family (e.g. they buy unhealthy foods, lack of acknowledgment on weight loss)  Inconvenient meeting times for support groups  Support group meetings being too far away or not having access to childcare (antecedent of social support) | Adherence  Social relationships | Participants shared their struggles with eating and exercise behaviors for weight maintenance. Common themes also included lack of support from family as well as lack of community support in the form of support groups. | N/A |
| Pereira da Silva & da Costa Maia, 2013 | Portugal | 30(20 women)  40.2 (±8.8)  Gastric banding and SG  1 year  Marital status, education and employment | Psychosocial  Technical and making sense  Physical  Making sense | The aesthetics of loose skin, guilt about not adhering to diet instructions  Complying with dietary advice and making lifestyle changes (such as eating behavior and physical exercise), having to sacrifice certain foods as they are unhealthy or do not work with the new stomach  Food tasting different after surgery  Being surprised and not having knowledge about certain aspects and outcomes after surgery | Adherence  Quality of life | Participants could be divided into equal groups of success and failure. In these groups, categories of outcomes and treatment emerged. Themes found within the success group included met expectations and lifestyle changes. The failure group emphasized lack of personal commitment and sacrifice. Further information about the process of treatment after bariatric surgery for patients is needed to ensure commitment post-surgery. | N/A |
| Geraci et al., 2014 | USA | 9(9 women)  42 (27-57)  SG and RYGB  2.5-7.5 years  Marital status and ethnicity | Technical  Physical  Psychosocial and mental | Adhering to dietary restrictions, taking supplements daily  Dumping syndrome and food intolerances  Grief in regard to not being able to enjoy the same meals socially | Adherence  Quality of life  Social relationships | Two main themes were found during the study; food in the year following bariatric surgery and bariatric surgery not being a magic pill. Participants found that after the first year after surgery, the work of adhering to diet and exercise becomes more difficult as weight loss slows down. | N/A |
| Lynch & Bisogni, 2014 | USA | 16(13 women)  No mean (32-62)  Gastric bypass  14 months - 10 years  Marital status, household comp, education, employment and education | Physical  Technical  Psychosocial, logistical and technical  Making sense | Not being able to eat more than a certain amount without negative consequences (such as dumping syndrome and food getting stuck). Low blood sugar. Food intolerances.  Adhering to and planning eating times, monitoring consumption of food through journals, preventing deficiencies through supplements, buying smaller sized portions and only eating half when eating out, dealing with cravings and preventing grazing  Fear of the stomach or pouch stretching, choosing not to drink when eating as to prevent this. Being surprised by still having to think about food all the time, specifically in regard to planning meals | Quality of life  Adherence | The main goals of patients were found to be: Weight management, overall health, avoiding negative reactions to eating, and integrating dietary changes with daily life. The monitoring and strategies used by patients illustrates the “work” put into adhering to a new lifestyle after surgery. | N/A |
| Groven et al., 2015 | Norway | 5(0 women)  No mean (24-46)  Gastric bypass  >4 years  Marital status and employment status | Physical  Psychosocial  Temporal, technical and logistical  Relational | Severe complications after surgery (internal bleeding, herniation and ulcers). Excess skin, the physical strain on the body when needing to remove excess skin surgically. Food restriction. Constipation. Hypoglycemia.  Struggling with excess skin and scars when meeting new partners or wearing shorts. Fear of losing one's job due to fatigue or sick leave. Fear of gaining weight again as it threatens the hegemonic masculinity that was enhanced after losing weight. Taking risks regarding food as to be seen as a “normal man” by others.  Finding ways of managing side-effects such as hypoglycemia by bringing certain foods and planning food intake. Lifestyle changes.  Doctors not taking one’s pain seriously. Relying on others. | Ability to work (fatigue and being on sick leave, after skin removal for example)  Quality of life (complications and side-effects)  Adherence (connected to masculinity)  Social roles (adhering to hegemonic masculinity and being a “normal man”) | The men related to their experiences after surgery in ambivalent ways. Relating to side-effects but also positive changes in ways that could be related to hegemonic masculinity. | Doctors not taking pain seriously and the men not expressing the intensity of their pain. Being stoic and enduring pain as traditional hegemonic masculinity norms.  Men being increasingly seen as responsible for the maintenance and appearance of their bodies  The insight of what is happening to the body as a threat and facilitator for masculine identity  Challenging traditional masculine norms by taking care of one’s health  Having to rely on others |
| Janse Van Vuuren et al., 2015 | Australia | 23(18 women)  Not reported  Gastric banding  Mean: 3.7 years  Marital status, education and employment status | Making sense  Physical  Relational  Technical and mental | Believing that surgery would be a quick fix and having to come to terms with the effort having to be put in after surgery  The restriction of the gastric band on food intake. The restriction leading to an increased desire for certain foods (like ice cream or chocolate)  The restriction of what one is able to eat and how that affects social interactions. Not attending dinners or socializing leading to isolation.  Restrictions leading to maladaptive eating and drinking patterns. Grief, loss, shame and loneliness leading to emotional eating. Struggling with not losing enough weight or gaining weight. | Quality of life  Worsening of symptoms (maladaptive eating behaviors)  Relationships  Adherence | Unrealistic expectations of the procedure was the core category found in the data. The surgery had a negative impact on the participants in the way that it restricted socializing and eating which led to maladaptive eating behaviors. | N/A |
| Groven, 2016 | Norway | 22(22 women)  No mean (24-54)  Gastric bypass  Not reported  Some information on marital status, education and employment status | Physical  Psychosocial  Psychosocial and mental  Technical  Making sense  Logistical and psychosocial | Dumping syndrome, fainting, feeling ill enough to have to lie down.  Experiencing cravings despite being full.  Having to ask their children for help with lying down. Feelings of guilt and anger after a dumping episode. Blaming oneself for the dumping episode. Fear of gaining weight.  Lifestyle changes to avoid dumping.  Feeling a sense of loss of control when not being able to adhere to lifestyle changes. The dissonance between the physical changes and restrictions leading to lifestyle changes, and the emotional as well as socially embedded previous habits. Thoughts about how the surgery does not magically fix disordered or emotional eating  Fear of dumping syndrome when at work or out in public. Trying to prevent dumping from happening in public to minimize the amount of people that know about the surgery. One woman’s fear of losing authority as a manager if people knew about the surgery. | Quality of life  Adherence  Ability to work  Social roles  Worsening of symptoms | Participants found that adjusting their food intake was specifically challenging after surgery. Personalized support is indicated to help aid individuals that are struggling with adhering to lifestyle changes after surgery. For this, it is necessary to thoroughly understand the challenges that come with adjusting food intake. | Understanding women’s experiences of life after bariatric surgery while being aware of how these experiences are embedded in a sociocultural world. Womanhood, what it means to be a woman.  Discussion about food and emotions as related to the feminine. Women are being expected to exercise control of their food intake. |
| Groven & Glenn, 2016 | Norway | 3(3 women)  No mean (28-50)  Not reported  >5 years  Marital status, education and employment status.  Co-morbidity of RA in one participant | Physical  Technical  Mental and Psychosocial and relational  Relational  Making sense | Dumping syndrome, hypoglycemia, internal herniation, fatigue, excess skin, dental issues  Lifestyle changes to minimize risk of dumping syndrome, hypoglycemia and maintain weight loss  Feelings of shame after gaining weight back. Struggling with other people's perceptions of weight gain. Feelings of guilt and being a terrible wife and mother due to being bedridden after surgical complications. Struggling with people’s questions about weight loss. Avoiding social interactions due to shame because of lack of success.  Disappointment in post-surgical care and followup in comparison to pre-surgical. Care mainly being monitoring and weighing, whereas emotional health is not being paid attention to. Asking for more care and support but not receiving it. Feeling judged by the care team regarding weight regain. Wishing for psychological follow-up care. Dissonance in professional’s recommendations and patient experiences.  Regretting the surgery. Not having received information about possible hypoglycemia. | Quality of life  Social roles and relationships  Worsening of symptoms  Adherence | Stories of those that have regained weight after WLS are often silenced and buried behind narratives of successful stories. Those that have regained weight may find it difficult to reveal their struggles and this can in turn increase their suffering. | Feelings of guilt in relation to being a wife and mother after suffering complications  “...large women find it difficult to see themselves in positive terms when others judgement keeps telling them otherwise” |
| Hillersdal et al., 2016 | Denmark | 24(15 women)  No mean (26-61)  RYGB  1.5-2 years  Occupation | Making sense  Technical | The surgery is not a cure for emotional or disordered eating. Old thought patterns and maladaptive eating pertaining.  Struggling to adhere to lifestyle changes, for example if dumping syndrome does not occur. |  | The three main categories found that describe patient’s coping strategies post-surgery are: surgery as time-out (the surgery facilitating lifestyle changes but not solving overweight in itself), surgery as a solution (the restriction on food intake) and strategy of abstaining (fear of negative side-effects) | N/A |
| Laurenius & Engström, 2016 | Sweden | 12(8 women)  47 (32-58)  RYGB  9 years | Physical  Logistical and temporal  Psychosocial  Making sense  Technical  Mental | Dumping syndrome  Having regular meals is important to avoid dumping syndrome. Also eating slowly. Contacting conferences or venues to let them know what one can eat.  Struggling at restaurants and social occasions such as holidays, as the food served is often fatty and triggering for dumping syndrome. Accepting dumping as a tool to not experience the stigma of being obese again. Trying to be okay with leaving food on the plate despite what others might think.  Viewing dumping syndrome as a positive consequence that prevents weight gain. Learning to deal with it.  Making lifestyle changes to avoid dumping syndrome  Blaming oneself for experiencing dumping syndrome |  | None of the participants saw dumping syndrome as a negative consequence, but more so a tool to prevent weight gain. Participants believed that dumping syndrome could mostly be controlled. | N/A |
| Liebl et al., 2016 | USA | 14(11 women)  47 (±12)  Gastric banding, gastric bypass and RYGB  >2 years  Race, marital status, education, employment, residence | Relational  Psychosocial  Mental  Technical  Making sense and mental  Relational, temporal and technical | The importance of support after surgery. Support from friends,  family and clinicians and how this can alter the effects of surgery (e.g. other family member’s altering their diet. Having to end relationships due to the negative impact they had post-surgery (one participant recalls her husband feeding her ice cream).  Some people undermine the efforts the patients go through after surgery to maintain weight. Hurtful comments. Trying not to let other people's opinions dictate choices about health. Struggling with the role of being a mom but also taking care of oneself. Using other people's opinions as a guide for maintenance weight.  Food addiction being replaced with other addictions. Still struggling with disordered eating.  Developing strategies and modifying eating behaviors that satisfy but do not compromise health.  Listening to the body’s fullness cues and tolerances. The mental changes needed to come to terms with the physical changes. Struggling to make sense of the new body, still seeing oneself as obese.  Having support means that patients were able to make time and engage in physical activities | Social roles and relationships  Adherence | The participants identified barriers and facilitators to maintaining their weight loss. Support from friends, family and professionals was one of the main findings that contributed to adherence to positive lifestyle changes. | Husbands having opinions on wives' choice to have the surgery.  Being a mom and struggling to put oneself first and take care of oneself. |
| Lier et al., 2016 | Norway | 10(7 women)  No mean (39-57)  Gastric bypass  5 years  Relationship, employment, plastic surgery | Psychosocial  Physical  Relational  Mental  Making sense  Technical  Temporal, logistical and making sense  Financial | Wanting to avoid stigma and caring about other people’s opinion about appearance. Worries that children will inherit their obesity and live with the challenges that entails. Feeling self-conscious when undressing in front of other people or being intimate (due to excess skin for example). Worries about sexual attractiveness. Struggling with eating in public as people question why food is left on the plate.  Excess skin. Having to tuck the skin into clothes. Dumping syndrome.  A new sense of self and changes in capability after surgery leading to relationships ending.  Fear of weight gain  Not being satisfied with weight loss after surgery, wishing to lose more. Some also expressed fear of losing too much and seeing one's bones.  Lifestyle changes in eating behavior, restrictions, changed rhythm. Exercise is still difficult in a smaller body and it is hard to find the motivation.  Taking time to learn how the new body works and how one can eat. Needing the ‘whole day’ to plan meals to be able to eat frequently, a big change from pre-surgery.  Being impulsive and spending a lot of money after surgery, as weight loss gave the experience of a ‘high’ | Quality of life  Social roles and relationships  Adherence | The participants describe major differences to their lives after undergoing bariatric surgery. This includes social relationships, changing lifestyle, their relationship to their changing body and self-esteem. |  |
| Lynch, 2016 | USA | 16(13 women)  No mean (32-63)  Gastric bypass  15 months - 10 years  Marital, household, education, employment, income | Technical  Making sense  Physical  Mental  Logistical  Temporal  Psychosocial and relational | Forming new habits and adhering to these when the maintenance period begins. Needing to take vitamin supplements. Trying to create good habits in the ‘honeymoon phase’ to adhere to in the ‘work’ phase of maintaining weight.  Cognitive and behavioral effort needed to adjust to managing weight and adapting to a new lifestyle. A trial and error period when participants figure out how the new body works, what foods they can tolerate and not, ‘retraining’ oneself how to eat. Having to adjust again some time after surgery when the physical restrictions no longer work the same, renewed cognitive effort. Listening to one's body but being aware of new eating strategies. Redefining the role of food after surgery, e.g. in relation to before surgery being an emotional eater. Surgery being viewed as a tool and still needing to put in work.  Physical restrictions of food intake, food intolerances, dumping syndrome, taste changes and aversions to textures. Changes in hunger signals.  Going to therapy and dealing with the re-emergence of emotional eating. The challenge of not being able to let one’s guard down and having to put work in for the rest of one’s life.  Eating protein first, measuring food, limiting carbohydrates.  Planning meals ahead, reading food labels, monitoring everything, eating slowly.  Big life events such as deaths in family and marriages impacting eating patterns and emotional eating. Maintaining lifestyle changes through marital problems, stress and holidays is viewed as an important aspect of weight management. | Adherence | Following patients after weight loss due to bariatric surgery, the researchers found paths of maintaining, regaining/losing, regained and losing. Dietary management components consisted of physical needs, hunger and fullness, relationship with food, strategy use, habit formation, and awareness of eating. The initial time after surgery was deemed easy as ‘the surgery does the job’ but after weight stabilization is when the ‘work begins.’ Differences in maintenance was associated with ability to maintain lifestyle changes, habits and awareness of eating behaviors. |  |
| Wood & Ogden, 2016 | UK | 10(9 women)  No mean (31-61)  VBG, LAGB and gastric bypass  >8 years  Ethnicity | Making sense  Mental  Technical | Sometimes forgetting to eat. Feeling like not being able to eat whatever is a type of deprivation. Functionalization of food, eating because you need it and not for an emotional gain. Still seeing oneself as a fat person.  In some cases, depression increased. Emotional eating is still present.  Creating physical and sedentary strategies to ignore thoughts of food. Making lifestyle changes. | Quality of life (mental)  Worsening of symptoms | The researchers found themes of eating behavior, relationship with food as well as quality of life. The differences between the participants within these themes (functionalizing food or not for example) were compatible differences in those who succeeded with weight loss and those who failed. | N/A |
| Liu & Irwin, 2017 | Canada | 28(21 women)  49.7 (±12.7)  Gastric bypass and gastric sleeve  >2 years  Ethnicity, marital status, professional status | Physical  Financial  Relational  Making sense  Technical  Mental and making sense | Excess skin leading to infections (that smell and itch) and it being uncomfortable to move and do sports which hinders physical activity.  Not being able to afford the treatment of excess skin removal.  Receiving mixed information regarding which types of plastic surgery are covered after weight loss. Wishing that more psychological support is offered and not only monitoring physical health and weight. Some partners being unsupportive and the surgery leading to divorce in some cases.  Re-learning how to eat and understanding what foods irritate the stomach. Getting used to the new body and how it looks. Still feeling like the largest person in the room.  Lifestyle changes and changing habits. Using a salad plate instead of a big dinner plate.  The excess skin leading to body dysmorphia. Wishing for psychological support such as counseling regarding body image after care at the clinic has concluded. Struggling with unrealistic expectations for surgery such as specific weight loss that is not achieved. | Quality of life  Social roles and relationships  Costs | Primary themes included physical changes and challenges (such as excess skin), psychological experiences (such as body dysmorphia), social support and clinic-related experiences and wishes (of more psychological support for example). | N/A |
| Lin & Tsao, 2018 | China | 17(11 women)  34.5  Gastric bypass and RYGB  12-35 months  Education, occupation, marital status | Technical and temporal  Making sense  Physical  Logistical  Relational and physical  Psychosocial  Mental | Lifestyle changes to minimize physical discomfort, such as smaller portions and eating slowly.  Not fully grasping the need for a change in eating habits after surgery and taking the initial weight loss for granted.  Nausea, vomiting, acid reflux, malnutrition (mainly related to not adhering to lifestyle changes compatible with bariatric surgery). Malnutrition leading to symptoms such as dizziness and shortness of breath.  Eating every 2-3 hours, eating protein first. Not adhering to follow-up visits, leading to malnutrition  Balancing relationships after surgery. Not taking advice regarding eating behavior from professionals leads to physical discomfort.  Finding it difficult to see family members eat foods that one cannot tolerate.  Changing feelings of hope, disappointment and failure | Quality of life  Adherence  Social roles and relationships | The researchers found two different living processes in the experiences of the participants; life modification or suffering. Not engaging in the life modification process leads to suffering. | N/A |
| Lloyd et al., 2018 | UK | 10(10 women)  No mean  (43-63)  LAGB  >2 years  Ethnicity, employment, marital status | Physical  Logistical  Making sense  Psychosocial  Mental  Relational | Side-effects of pain, regurgitation and vomiting. Food getting stuck and causing discomfort.  Always looking for where the nearest toilet is in case of becoming sick.  Not knowing beforehand about uncomfortable side-effects. Consuming the “wrong” foods such as chocolate as it was sometimes easier to stomach than a salad.  Fear of vomiting leading to avoiding social interactions and isolation.  Feelings of regret and disappointment after the surgery and gaining weight again.  Not receiving enough support and information after the surgery from professionals. Wishing for more mental support (in regard to previous disordered eating for example) in combination with the physicality of surgery. Wanting life-long support from psychologists and support groups for the mental effects. | Quality of life (physical, mental, social)  Adherence  Social roles and relationships | Three major themes emerged: living with the side effects of having surgery (such as pain and vomiting), regret due to side effects and weight regain and lack of support from caregivers after surgery. | N/A |
| Spadola et al., 2018 | USA | 12(10 women)  28.2 (±4.5)  RYGB and LSG  5 - 79 months  Ethnicity | Physical  Making sense  Mental | Increased alcohol sensitivity.  Not having knowledge about the increased alcohol sensitivity.  Using alcohol to cope with stress as it is more difficult to use food for emotional eating after surgery. Being frustrated about what one cannot eat and using alcohol instead. Using alcohol as a coping mechanism for not losing weight. |  | Researchers found four main themes relating to the increased use of alcohol after bariatric surgery; increased sensitivity to alcohol, using alcohol as a coping mechanism, using alcohol as replacement for food and increased socialization. | N/A |
| Kabu Hergül & Özbayır, 2021 | Turkey | 32(26 women)  40.3 (20-56)  Not reported  Not reported | Technical  Making sense  Physical  Mental  Psychosocial  Relational | Creating new eating habits, eating less, more slowly, chewing more.  Adapting to the decreased volume of the stomach. Still wanting to eat more despite feeling full. Struggling to adapt to the new body. Initially regretting the surgery.  Feeling a pressure in the chest when eating too much. Constipation, stomach pains, dizziness, malnutrition, hair loss. Excess skin.  Fear of wound dehiscence, leakages of anastomosis, enlarging the stomach, weight gain and death.  Not feeling comfortable or beautiful when being naked due to excess skin.  Unsupportive friends and family. Wishing for more support from care providers such as psychological support, more information about diet and physical activity. | Quality of life  Adherence | In this paper, several physiological, psychological and social changes and challenges were found within the experiences of the participants. Adherence to lifestyle changes and social support could alleviate some of these challenges. | Not feeling beautiful with the excess skin |
| Reaves et al., 2019 | UK | 14(9 women)  Not reported  RYGB and LSG  Mean: 8.83  /5.06 | Mental  Physical  Psychosocial  Relational  Making sense | Using alcohol to cope with the restrictions of surgery or as a transfer from previous emotional eating. Fear of alcohol becoming an issue. Feeling shame about weight regain and using alcohol to cope. Feeling deprived of the positive emotional experience from eating certain food.  Increased sensitivity to alcohol. Physical restrictions in eating behavior. A sweet treat causing pain but a bottle of wine working well with the new body after surgery. Dumping syndrome.  Struggling with the fact that one has to eat smaller portions when eating socially and instead using alcohol to fill the gap.  Lack of emotional support post-surgery or not seeking out support. Disliking the time-limited nature of the instrumental support from care teams (the support stopping after 2 years for example).  Not feeling prepared enough and struggling with adapting to lifestyle changes and psychological changes after surgery. Not receiving information regarding the change in alcohol sensitivity after surgery. | Quality of life  Adherence  Social roles and relationships | Themes that were found from the experiences of participants both with and without problematic alcohol use were: drinking motivations, self-image, impact of restriction on eating behavior, support needs and surgical preparedness. Those with problematic alcohol use seemed to struggle more with all aspects of the themes. | N/A |
| Watson et al., 2020 | UK | 5(5 women)  37.6  (29-55)  RYGB  9 months - 8 years  Ethnicity, relationship status, employment | Mental  Psychosocial  Physical | Fear of regaining weight lost after surgery and engaging in restrictive eating. Struggling with the look of excess skin. Feeling like one would be more confident in weight loss without the excess skin. Feeling guilty about enjoying food.  Weight loss not being visible due to excess skin. The aesthetics of excess skin reminds the participants about their previous obese bodies.  Excess skin | Quality of life | Themes of weight stigma, the impact of loose skin and thoughts about food and disordered eating patterns contributed to restrictive eating disorders in women after bariatric surgery. | Psychological issues and body image of women after bariatric surgery. Previous stigma and bullying contributed to fear of gaining weight after surgery. |
| Yu et al., 2020 | China | 15(5 women)  38.5  (±14.4)  LSG  13 - 75 months | Physical  Psychosocial  Technical  Making sense  Logistical  Temporal | Nausea, hypoglycemia, difficulty swallowing and reflux. Hair loss and loose skin.  The appearance of hair loss and loose skin. Social events can be awkward because one is not able to eat as much as everyone else. Limited choice of restaurants because of this (preferring hotpots where people share food and talk a lot). Avoiding follow-up appointments due to crowded hospitals, not wanting to lose blood and gastroscopies being uncomfortable.  Lifestyle changes such as eating behavior. Struggling with motivation for exercise.  The journey of getting to know the new body. Experiencing discomfort when eating certain foods and adjusting habits accordingly.  Monitoring weight and adjusting food intake and physical activity accordingly.  Avoiding follow-up appointments as it takes a long time to see one's doctor. Not having enough time for exercise. | Quality of life  Social roles and relationships  Adherence | Two of the identified major themes include self-management strategies (such as lifestyle changes in the form of eating behavior) and culture-specific difficulties in regard to adherence to lifestyle changes and follow-up visits. | N/A |
| Tolvanen et al., 2021 | Sweden | 16(12 women)  49  Gastric bypass and sleeve gastrectomy  Not reported | Relational  Mental  Psychosocial  Making sense  Relational and temporal | Insufficient support from care providers that lacked important components (such as information about adherence to diet and portion sizes). Feeling responsible for seeking out support but being uncertain about where to turn. Insufficient communication between medical institutions. Follow-up visits focusing too much on measurements and not psychological well-being after surgery.  Lack of support leading to increased feelings of shame, sadness and abandonment. Not seeking help when experiencing weight gain because of self-blame and shame. Using alcohol instead of emotional eating when struggling psychologically.  Support from friends and family sometimes being stigmatizing and discouraging. Receiving negative remarks from others regarding weight regain.  Information about surgery is difficult to understand and varies between clinics. Having unrealistic expectations and having to come to terms with reality. Not having received enough information about possible weight regain and the importance of adhering to dietary guidelines. Wishing for life-long individualized support from professionals to prevent weight regain or alcohol abuse for example.  The importance of support from family and partners to be able to sit down and have slow meals. | Social roles and relationships | The two main themes found in relation to weight regain after bariatric surgery were; a lonely struggle and others as a source of compassion and control. Lacking support from care providers and sparse follow up visits were described to contribute to difficulties in maintaining weight. | Implicit: needing the support of a partner to help with the household |

**Appendix 2. Extraction table for papers included from additional search**

| **Paper** | **Country** | **Ant: Sex, x̅ age (range), surgery, time since** | **Type of TB attributes** | **Aspects of TB attributes** | **Cons.** | **Main findings** | **Gender diff** |
| --- | --- | --- | --- | --- | --- | --- | --- |
| Lindberg et al., 2022 | Sweden | 16(8 women)  No mean  (29-54)  -  >4 years  Education, marital status | Physical  Psychosocial  Making sense  Mental | Excess skin makes it difficult to perform physical activity.  Not wanting to be naked due to the excess skin, for example in a public bath. The new body warranting unwanted and intrusive questions from others. Reduced sexual pleasure from not feeling “special” and chosen anymore.  Getting used to a new smaller body, missing the safety of the old body. Still seeing the old bigger body in the mirror.  Not being able to use food as an emotional escape anymore and developing transfer addictions to alcohol for example. | Quality of life  Social roles and relationships | Despite experiencing improved health after undergoing bariatric surgery, participants also struggled with getting used to the new body (both mentally and physically) as well as other people’s reactions to it. | N/A |
| Tettero et al., 2022 | Netherlands | 18 patients(14 women)  48 (21-65)  RYGB and SG  - | Making sense and mental  Technical | Struggling to come to terms with suboptimal weight loss. Emotional reactions to being invited for an intervention regarding suboptimal weight loss, feeling like a failure  Adjusting bad eating habits, planning physical activities | Social roles and relationships (to physicians)  Adherence | A barrier to patient participation in interventions for suboptimal weight loss after bariatric surgery was found to be the patient's emotional response to confrontation from physicians. A facilitator found was the respect of autonomy from physicians. | N/A |
| Billing-Bullen et al., 2022 | New Zealand | 28(20 women)  49.7 (±8.8)  Gastric sleeve  >12 months  Ethnicity | Physical  Mental  Financial  Technical  Making sense  Temporal  Psychosocial  Relational | Excess skin. Food intolerances with side-effects such as nausea, vomiting and reflux.  Fear of gaining weight again. Decrease in self-confidence due to excess skin. Still feeling mental hunger despite being full or just having eaten. Still struggling with binge-eating and emotional eating. Feeling fat. Obsessing about mealtimes and what to eat.  Purchasing nutritious food is deemed expensive.  The challenge of adhering to lifestyle changes such as changing eating behavior.  Not being knowledgeable about healthy food and cooking.  Time limitations when it comes to making good food decisions and engaging in physical activity  “All participants noted an increased awareness of food being the centre of many social activities, work meetings, and cultural celebrations, and felt they were missing out due to the required eating habit changes” Not telling people about the surgery due to fear of judgment and criticism.  Feeling under-supported by the bariatric team after surgery. “Being left to your own devices.” Wishing for longer follow-up care beyond 12 months. “In hindsight, participants expressed a need for more education and support pre-surgery, regarding diet, psychological and mental support.”  Follow-up stopping when most support is needed, to stabilize weight and maintain weight. | Quality of life  Social roles and relationships  Adherence  Costs | Some of the main findings of challenges include; managing a new healthy lifestyle (such as financial stress or challenging social events), changing eating behavior, mindset changes and on-going support.  SEE RECOMMENDATIONS | N/A |
| MacAskill et al., 2023 | Australia | - | Technical  Temporal  Relational  Psychosocial  Mental  Making sense | Creating new healthy habits when the initial “easy” weight loss is happening to maintaining these habits when the “work begins” to stabilize weight.  Meal planning, meal frequency, meal duration.  Decline in support from friends and family when weight stabilizes.  Finding additional attention and comments about weight to be uncomfortable. Decreased sexual confidence and shame about excess skin.  Fear of weight gain and the stress of excess skin. The shame of weight regain leading to avoidance of follow-up appointments.  Internal vs external locus of control in regard to weight maintenance after surgery. | Quality of life  Social roles and relationships  Adherence | There were three main themes of changing food relationships, navigating inter- and intrapersonal influences and caring health professionals. | N/A |
| Er et al., 2023 | Belgium | - | Mental  Physical  Relational | Body dysmorphia, still feeling fat when looking in the mirror despite weight loss. An initial “honeymoon” period after rapid weight loss was followed by an onset of old or new psychological problems. Using alcohol to cope with stress and the emotions of not losing weight. Alcohol replacing food as a coping mechanism after bariatric surgery. Frustration regarding not being able to eat certain foods as they cause discomfort.  Physical discomfort after eating. Dumping syndrome. Increased sensitivity to alcohol.  “Participants also recommended post operative counselling, as well as the need for long-term support from the bariatric team.” Wishing for longer term follow up and more information about the effects of alcohol after bariatric surgery. Wishing for more psychological support. | Quality of life (physical and mental)  Adherence | Persistence and reappearance of psychological problems after bariatric surgery, using alcohol as a coping mechanism and replacement for food, changes in physiological reaction to alcohol and the importance of information regarding the risks of alcohol use after bariatric surgery were the synthesized results in the study. | N/A |
| Ansari & Serjeant, 2023 | UK | - | Physical  Mental  Technical  Making sense  Psychosocial | Physical restrictions within the gastrointestinal system leading to dumping syndrome, vomiting and reflux.  Psychological stress due to food choices being severely restricted. Triggering regret in having the surgery. Psychological hunger, wanting to eat despite being full.  Making changes to eating behavior to avoid unwanted side-effects. Some felt post surgery diet recommendations were excessive, burdensome and deviated from a normal way of eating, leading to selective adherence to healthy eating practices in some cases  Figuring out the new body, what food and portion sizes that work. Adapting to the look of the new body.  Body dysmorphia due to excess skin, avoiding social situations. Stigmatization of the choice of going through bariatric surgery. Losing weight through surgery is deemed the “wrong way.” Feeling chastised when eating small portions or not being able to finish a meal in social situations. | Quality of life  Adherence  Social roles and relationships | Four themes were identified during analysis including; relationship with food, relationship with oneself, relationship with others and unfinished journey. | N/A |
| Klapsas & Hindle, 2023 | Australia | 17(17 women)  30.5 (±13.1)  SG, gastric banding, RYGB, gastric bypass | Technical  Making sense  Temporal and logistical  Mental | Lifestyle change, eating behavior.  Learning to trust the body, it’s new signals and intuitive eating  Setting a timer when eating, specific allocated eating times, having enough protein in a meal, set portions.  Still struggling with a diet mindset, “bad” food, restrictions. Fear of the sleeve stretching if not eating correctly. | Quality of life  Adherence | Post-surgery dieting was experienced among the participants. A lifestyle change that included both restriction due to surgery but also flexibility and intuitive eating was an overarching theme. The descriptions of lifestyle changes after surgery often mirrored pre-surgery dieting. | Only women responded. Diet culture and women. |
| Funk et al., 2023 | USA | 24 patients(20 women)  50.6 (±10.7)  SG and RYGB  >1 year  Race, ethnicity, insurance status, marital status, education, work status, household income, financial situation. | Logistical, temporal and technical  Financial  Logistical  Making sense | Patient occupation interfering with adhering to post-surgery guidelines. Physically demanding jobs, multiple jobs, jobs that require traveling; difficult to plan meals. Working nights and not being able to join support groups. Sedentary jobs or working from home makes it difficult to adhere to guidelines on physical activity.  Not being able to afford nutritious food, vitamins and supplements.  Not having a car or reliable transport makes it difficult to attend follow-up visits. Living in a rural area makes it more difficult to access nutritious foods as convenient stores are more frequent.  Lack of health literacy making it difficult to adhere to post-surgery guidelines. | Adherence  Costs | Struggles and barriers to weight loss after bariatric surgery included: challenging employment situations, limited income, unreliable transportation, unsafe/inconvenient neighborhoods, health literacy. Interventions that target socioeconomic differences are needed to support patients after bariatric surgery. | N/A |
| Tolvanen et al., 2023 | Sweden | 16(12 women)  49 (20-64)  Gastric bypass  3-15 years  County of birth, employment, civil status | Making sense  Technical  Temporal  Psychosocial  Physical  Mental  Relational | Not being prepared for new obstacles such as maintaining weight and new effects of alcohol and food. Blaming oneself for struggling to adhere to lifestyle changes. Learning about the new body and food tolerance. Limited knowledge about dietary patterns and the increased effect of alcohol.  Struggling to adhere to regular meal patterns. Skipping meals.  The frequency and duration of meals being stressful with young children  Eating with family instead of keeping a meal structure. Social eating is viewed as challenging as it often leads to consumption of high-energy-dense food and drinks. Fear of dumping and early satiety when eating in social situations, reducing the enjoyment of food.  Certain foods cause reactions such as constipation, diarrhea and nausea. Dumping syndrome and the following fatigue.  Struggling with emotional eating and disordered eating (since before surgery). Alcohol abuse to deal with negative emotions.  Not receiving support from professionals when gaining weight after surgery. Information before surgery mainly included information about the procedure and dietary guides for the period after, not long term. No psychological support and insufficient nutritional support after the initial postoperative period. | Quality of life  Adherence  Social roles and relationships | “Some patients with weight regain were exposed to over- whelming dietary challenges following gastric bypass surgery. They were unprepared for the struggle with eat- ing habits, cravings, and emotional and disordered eating behaviors. Furthermore, they lacked sufficient nutritional knowledge, support, and adequate behavioral tools to resist weight regain.” | N/A |
| Graham et al., 2023 | UK | 14 patients(11 women)  50 (26-70)  RYGB, gastric bypass  7 months - 6 years    Ethnicity | Relational  Mental | Patients report that general practitioners do not seem to have an understanding of what those who have had bariatric surgery go through. Having to ask for yearly blood work to be done. Wishing for longer term support and more mental support (emphasized several times). Wanting more support from professionals regarding maintaining a proper diet after surgery.  Mental suffering from the changes after surgery, changes to the body and relationships. | Quality of life (mental)  Adherence | The lack of a framework for long-term follow up care after bariatric surgery was deemed as frustrating. Increased support regarding diet as well as psychological support was a wish among participants. | N/A |
| Bi et al., 2024 | China | -  See meta-integration for quantitative studies for antecedents | Mental  Making sense and technical  Financial  Temporal | Negative attitude and its impact on dietary compliance.  Lack of awareness, access to information and understanding of postoperative dietary adherence  The cost of postoperative supplements  Complex dosing schedules of supplements. |  |  |  |
| Jawara et al., 2024 | USA | 24 patients(20 women)  50.6 (±10.7)  SG and RYGB  >1 year  Ethnicity, insurance,edu, empl, household income, marital status | Technical and psychosocial  Logistical and technical  Logistical and relational | Difficult to adhere to lifestyle changes required after bariatric surgery when being part of a family that encourages finishing every meal.  Planning to walk and exercise with others to keep oneself accountable  Struggling to get to follow-up appointments when living in rural areas due to fear of driving or lack of public transportation. Needing friends and family to drive to appointments. | Social roles and relationships  Adherence | Four themes were identified regarding the effect of social support on weight loss after bariatric surgery: family involvement in adjusting to the new diet, engaging in activities with partners and friends, help with transportation to appointments, life stressors experienced within relationships. | N/A |

**Complete reference list (in order of publication year, ascending order)**

1. Engström M, Forsberg A. Wishing for deburdening through a sustainable control after bariatric surgery. Int J Qual Stud Health Well-being. 2011 Feb 15;6(1).
2. Benson-Davies S, Davies ML, Kattelmann K. Understanding Eating and Exercise Behaviors in Post Roux-en-Y Gastric Bypass Patients: A Quantitative and Qualitative Study. Bariatr Surg Pract Patient Care. 2013 Jun;8(2):61-68.
3. da Silva SS, da Costa Maia Â. Patients' experiences after bariatric surgery: a qualitative study at 12-month follow-up. Clin Obes. 2013 Dec;3(6):185-93.
4. Geraci AA, Brunt A, Marihart C. The Work behind Weight-Loss Surgery: A Qualitative Analysis of Food Intake after the First Two Years Post-Op. ISRN Obes. 2014 Jan 9;2014:427062.
5. Lynch A, Bisogni CA. Gastric bypass patients' goal-strategy-monitoring networks for long-term dietary management. Appetite. 2014 Oct;81:138-51.
6. Groven KS, Galdas P, Solbrække KN. Becoming a normal guy: Men making sense of long-term bodily changes following bariatric surgery. Int J Qual Stud Health Well-being. 2015 Dec 4;10:29923.
7. Janse Van Vuuren M, Strodl E, White KM, Lockie P. Psychosocial presentation of revisional LAGB patients: a qualitative study. Clin Obes. 2015 Oct;5(5):273-80.
8. Groven KS. "Then I Can Become Very Ill": Women's Experiences of Living With Irreversible Changes in Their Viscera. Health Care Women Int. 2016 Jun;37(6):599-619.
9. Groven KS, Glenn NM. The experience of regaining weight following weight loss surgery: A narrative-phenomenological exploration. Health Care Women Int. 2016 Nov;37(11):1185-1202.
10. Hillersdal L, Christensen BJ, Holm L. Patients' strategies for eating after gastric bypass surgery: a qualitative study. Eur J Clin Nutr. 2016 Apr;70(4):523-7.
11. Laurenius A, Engström M. Early dumping syndrome is not a complication but a desirable feature of Roux-en-Y gastric bypass surgery. Clin Obes. 2016 Oct;6(5):332-40.
12. Liebl L, Barnason S, Brage Hudson D. Awakening: a qualitative study on maintaining weight loss after bariatric surgery. J Clin Nurs. 2016 Apr;25(7-8):951-61.
13. Lier HØ, Aastrom S, Rørtveit K. Patients' daily life experiences five years after gastric bypass surgery--a qualitative study. J Clin Nurs. 2016 Feb;25(3-4):322-31.
14. Lynch A. "When the honeymoon is over, the real work begins:" Gastric bypass patients' weight loss trajectories and dietary change experiences. Soc Sci Med. 2016 Feb;151:241-9.
15. Wood KV, Ogden J. Patients' long-term experiences following obesity surgery with a focus on eating behaviour: A qualitative study. J Health Psychol. 2016 Nov;21(11):2447-2456.
16. Liu RH, Irwin JD. Understanding the post-surgical bariatric experiences of patients two or more years after surgery. Qual Life Res. 2017 Nov;26(11):3157-3168.
17. Lin HC, Tsao LI. Living with my small stomach: The experiences of post-bariatric surgery patients within 1 year after discharge. J Clin Nurs. 2018 Dec;27(23-24):4279-4289.
18. Lloyd FMM, Hewison A, Efstathiou N. "It just made me feel so desolate": Patients' narratives of weight gain following laparoscopic insertion of a gastric band. J Clin Nurs. 2018 Feb;27(3-4):732-742.
19. Spadola CE, Wagner EF, Varga LM, Syvertsen JL, De La Cruz Munoz NF, Messiah SE. A Qualitative Examination of Increased Alcohol Use after Bariatric Surgery among Racially/Ethnically Diverse Young Adults. Obes Surg. 2018 Jun;28(6):1492-1497.
20. Kabu Hergül F, Özbayır T. I Am As Normal As Everyone Now. . . : Examination of Experiences of Patients Undergoing Bariatric Surgery According to Roy's Adaptation Model: A Qualitative Study. Clin Nurs Res. 2021 May;30(4):511-521.
21. Reaves DL, Dickson JM, Halford JCG, Christiansen P, Hardman CA. A Qualitative Analysis of Problematic and Non-problematic Alcohol Use After Bariatric Surgery. Obes Surg. 2019 Jul;29(7):2200-2209.
22. Watson C, Riazi A, Ratcliffe D. Exploring the Experiences of Women Who Develop Restrictive Eating Behaviours After Bariatric Surgery. Obes Surg. 2020 Jun;30(6):2131-2139.
23. Yu Y, Burke LE, Shen Q, Imes CC, Sun R, Groth S, Zhang W, Kalarchian MA. A Qualitative Exploration of Patients' Experiences with Lifestyle Changes After Sleeve Gastrectomy in China. Obes Surg. 2020 Aug;30(8):3127-3134.
24. Tolvanen L, Svensson Å, Hemmingsson E, Christenson A, Lagerros YT. Perceived and Preferred Social Support in Patients Experiencing Weight Regain After Bariatric Surgery-a Qualitative Study. Obes Surg. 2021 Mar;31(3):1256-1264.
25. Lindberg S, Wennström B, Larsson AK. Facing an unexpected reality - oscillating between health and suffering 4-6 years after bariatric surgery. Scand J Caring Sci. 2022 Dec;36(4):1074-1082.
26. Tettero OM, Westerman MJ, van Stralen MM, van den Beuken M, Monpellier VM, Janssen IMC, Steenhuis IHM. Barriers to and Facilitators of Participation in Weight Loss Intervention for Patients with Suboptimal Weight Loss after Bariatric Surgery: A Qualitative Study among Patients, Physicians, and Therapists. Obes Facts. 2022;15(5):674-684.
27. Billing-Bullen G, Nielsen D, Wham C, Kruger R. Enablers and barriers to prevent weight-regain post bariatric surgery - A qualitative enquiry. Eat Behav. 2022 Dec;47:101677.
28. MacAskill W, Gillanders T, Wylie N, Pinidiyapathirage J. Finding what works-Patients' long-term experiences of weight maintenance post bariatric surgery: A systematic review and thematic synthesis of qualitative studies. Obes Rev. 2023 Nov;24(11):e13608.
29. Er E, Durieux N, Vander Haegen M, Flahault C, Etienne AM. Patients' perceptions of the mechanisms underlying alcohol use problems after bariatric surgery: A qualitative systematic review. Clin Obes. 2023 Feb;13(1):e12551.
30. Ansari M, Serjeant S. Patient experiences of weight loss and eating after bariatric surgery: A systematic review and qualitative synthesis. J Hum Nutr Diet. 2023 Aug;36(4):1438-1450.
31. Klapsas M, Hindle A. Patients' Pre and Post-Bariatric Surgery Experience of Dieting Behaviours: Implications for Early Intervention. Obes Surg. 2023 Sep;33(9):2702-2710.
32. Funk LM, Alagoz E, Murtha JA, Breuer CR, Pati B, Eierman L, Jawara D, Farrar-Edwards D, Voils CI. Socioeconomic disparities and bariatric surgery outcomes: A qualitative analysis. Am J Surg. 2023 Apr;225(4):609-614.
33. Tolvanen L, Christenson A, Bonn SE, Surkan PJ, Lagerros YT. Patients' Perspectives on Dietary Patterns and Eating Behaviors During Weight Regain After Gastric Bypass Surgery. Obes Surg. 2023 Aug;33(8):2517-2526.
34. Graham Y, Fox A, Mahawar K, Parrott J, Khalil F, Hayes C. Developing a long-term follow up service for bariatric surgical patients in the community: Patient and professional perspectives. Obes Sci Pract. 2023 Jan 12;9(4):346-354.
35. Bi Y, He L, Yan F, Liu Y, Zhang Y, Gong R. Personal, external, and psychological factors influencing adherence to nutrition and diet in patients undergoing metabolic/bariatric surgery: a systematic synthesis of mixed methods research. Acta Diabetol. 2024 Sep;61(9):1083-1095.
36. Jawara D, Alagoz E, Lauer KV, Voils CI, Funk LM. Exploring Social Support Dynamics After Bariatric Surgery: Insights From Patients and Providers. J Surg Res. 2024 Jul;299:1-8.
